# 193. Rapid Diagnostic Testing and Vancomycin Utilization for Contaminated Blood Cultures

**DOI:** 10.1093/ofid/ofac492.271

**Published:** 2022-12-15

**Authors:** Connor Lockridge, Kristen Paciullo, Ronald Trible

**Affiliations:** Emory Saint Joseph's Hospital, Atlanta, Georgia; Emory Saint Joseph's Hospital, Atlanta, Georgia; Emory Saint Joseph's Hospital, Atlanta, Georgia

## Abstract

**Background:**

Bacteremia is a prevalent hospital acquired infection that affects nearly one in ten hospitalized patients. Rates of contaminated blood cultures have ranged from 0.6% to 6% in the literature. Vancomycin is often chosen as empiric coverage for gram positive organisms while awaiting bacterial identification. The purpose of this study was to evaluate the impact of GenMark ePlex Blood Culture Identification Panel (BCID) implementation on the duration of vancomycin therapy for blood cultures contaminated with coagulase negative staphylococci (CoNS).

**Methods:**

A retrospective chart review was conducted on patients meeting inclusion criteria during a six month period prior to BCID implementation and post BCID implementation, from 03/01/2019 to 08/31/2019 and 03/01/2021 to 08/31/2021. The primary outcome analyzed was the duration of vancomycin therapy, in hours, in patients with a contaminated blood culture. Contaminated blood cultures was defined as a single blood culture growing CoNS. Secondary outcomes included hospital length of stay, readmission for treatment of contaminated blood cultures, and nephrotoxicity.

**Results:**

This IRB-approved study included 190 patients, 75 in the pre-BCID group and 115 in the post-BCID group. The average duration of vancomycin therapy was 53.7 hours in the pre-BCID group and 20.5 hours in the post-BCID group (p = 0.04). Time to organism identification was reduced from 22.8 hours in the pre-BCID group to 12 hours in the post-BCID group (p = 0.5). Additionally, our study did not find a difference in nephrotoxicity, hospital length of stay, or readmissions between the two groups.

**Conclusion:**

Following implementation of the GenMark ePlex Blood Culture Identification Panel (BCID), there was a reduction in both duration of vancomycin therapy and time to culture identification for blood cultures contaminated with CoNS. Rapid diagnostic testing is helpful in clinical decision-making and can lead to earlier discontinuation of empiric antibiotics, such as vancomycin.

**Disclosures:**

**All Authors**: No reported disclosures.

